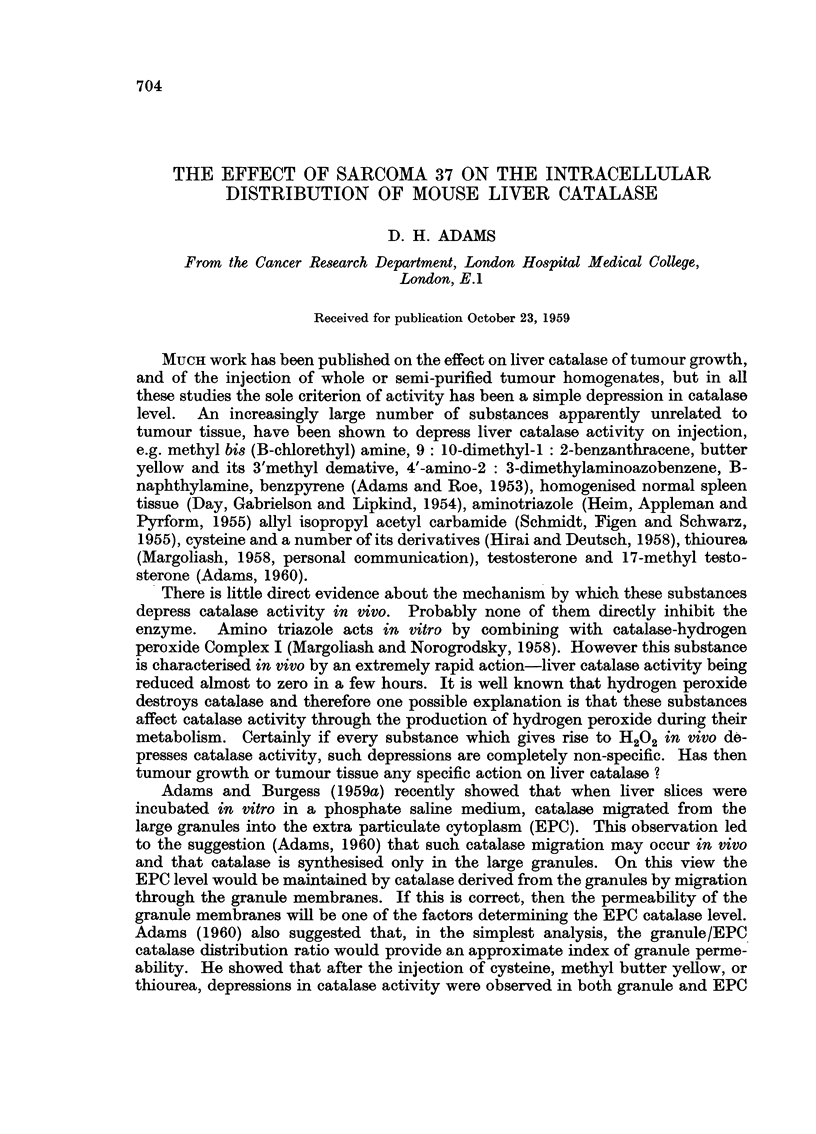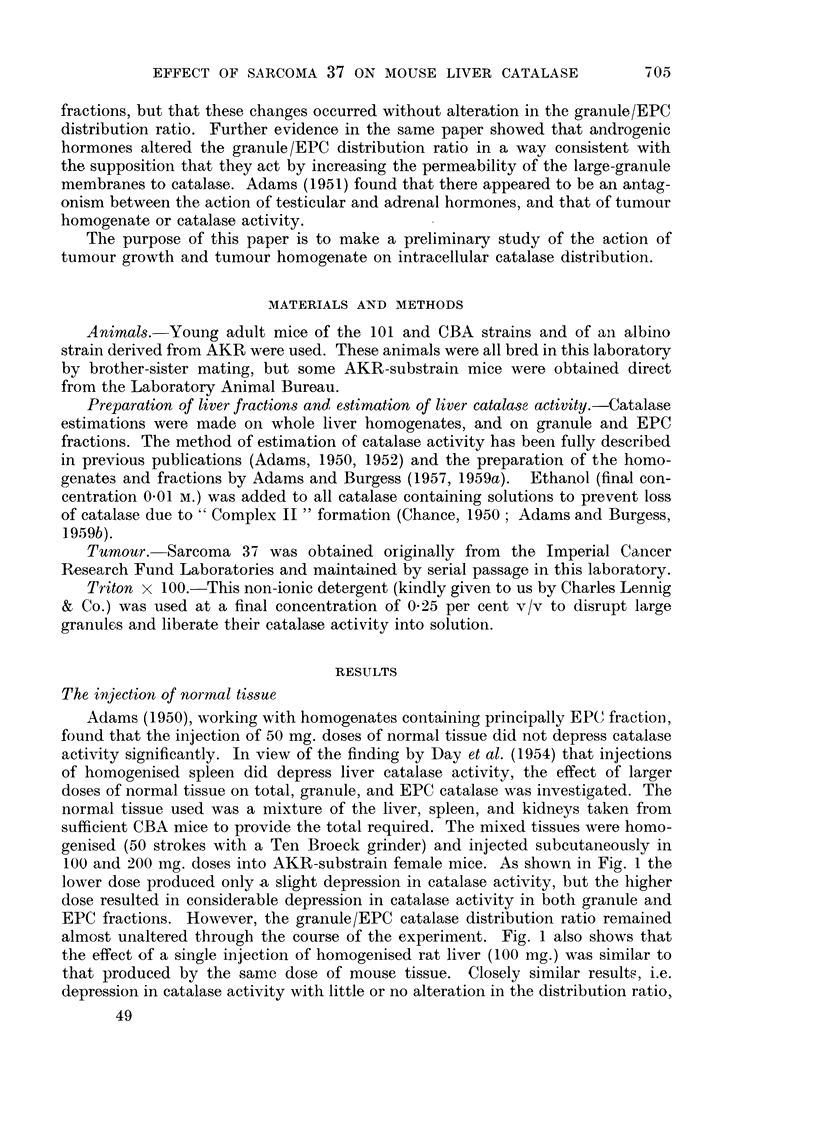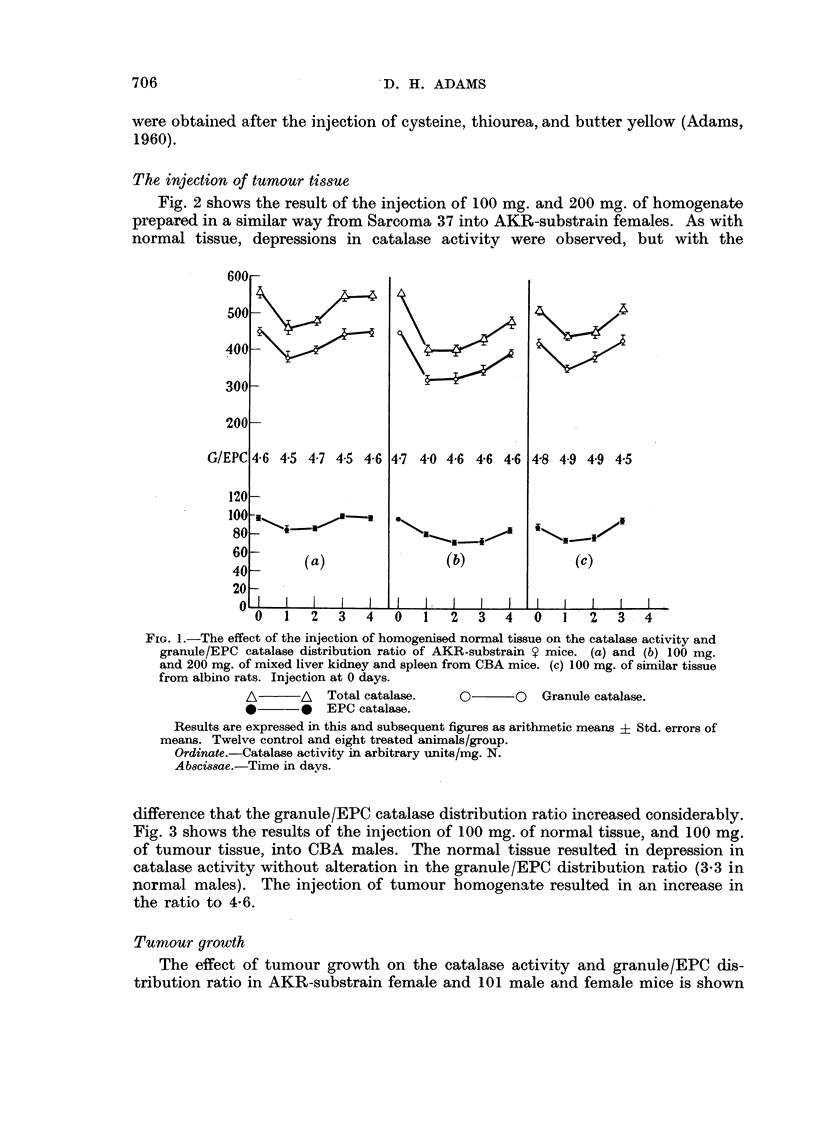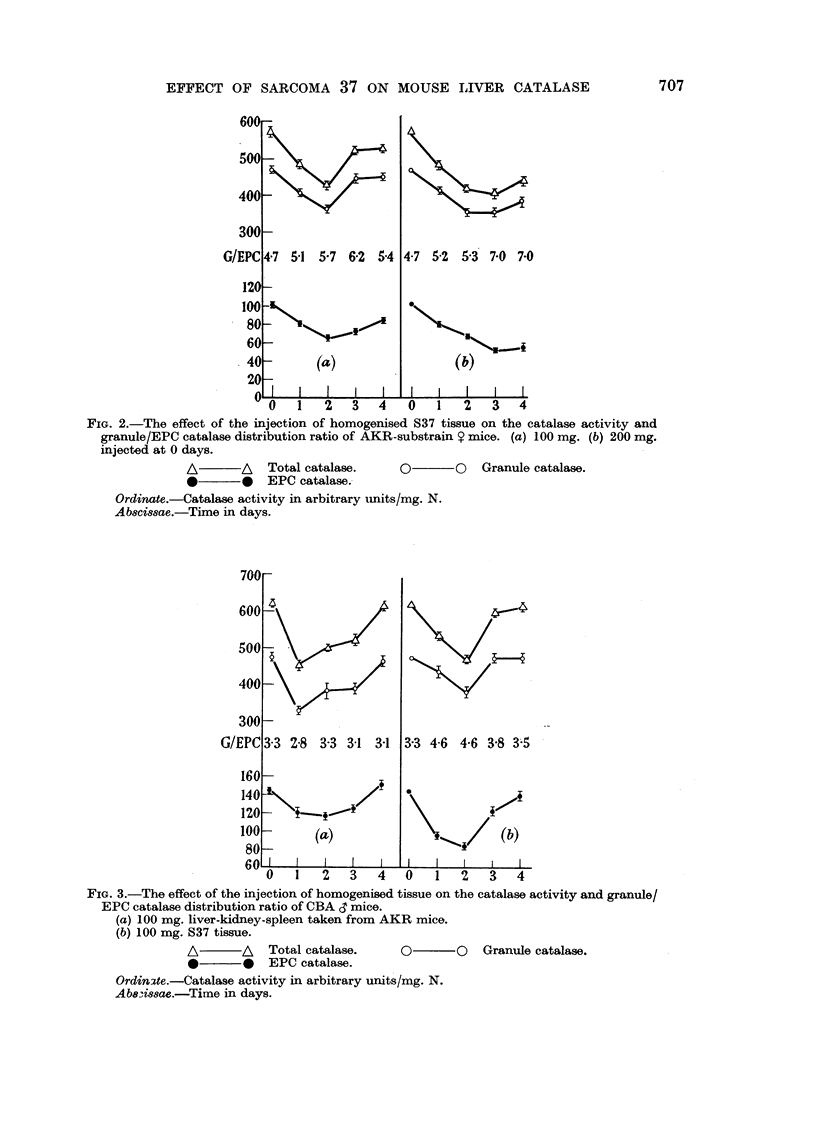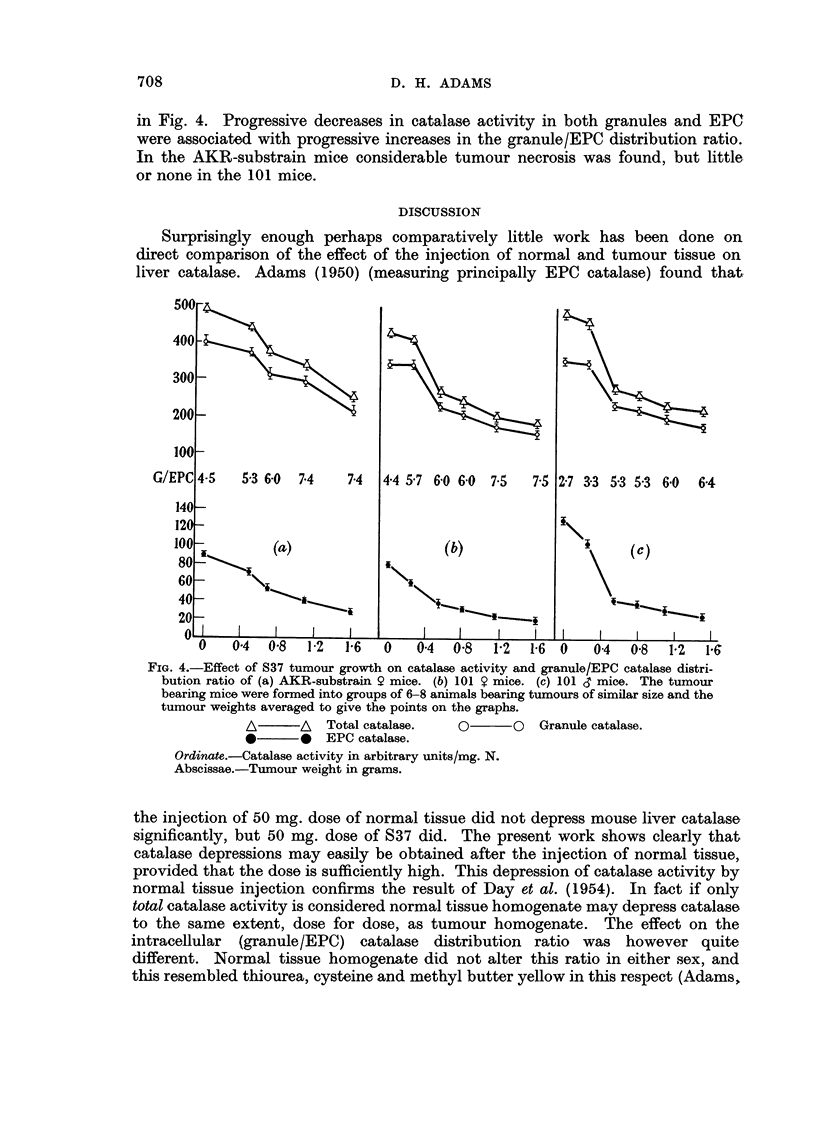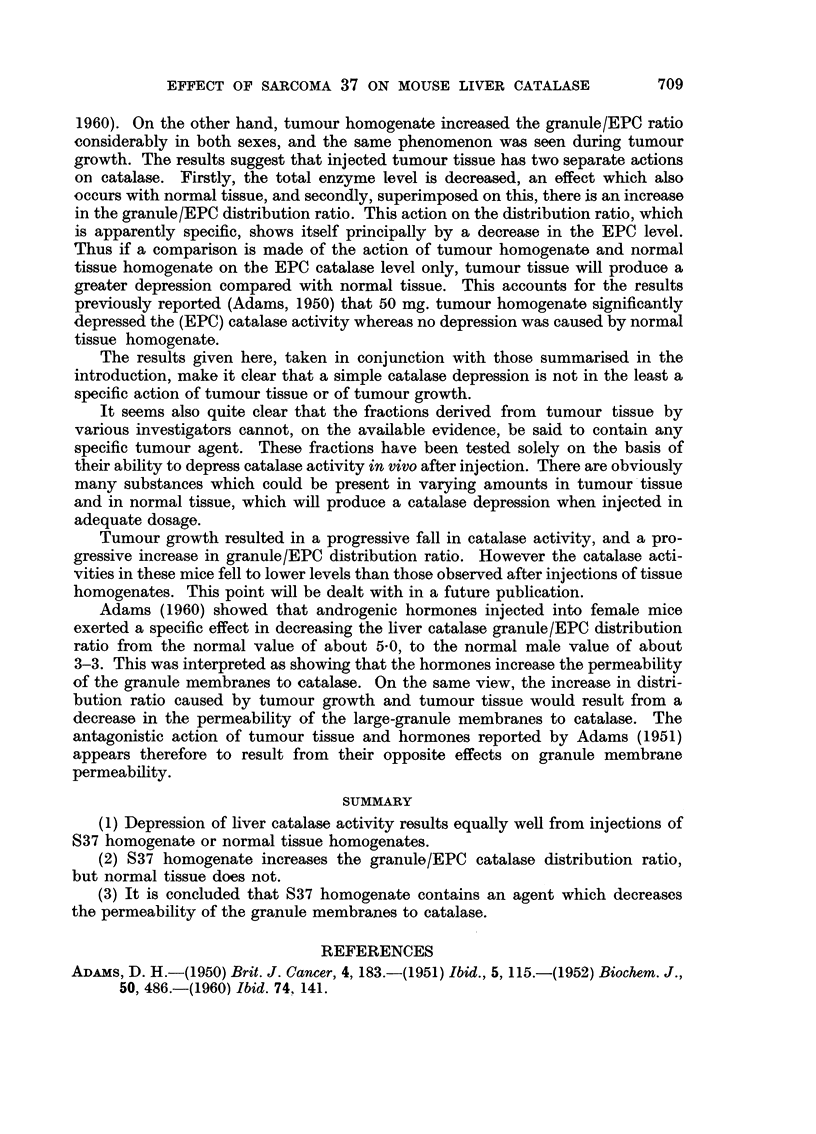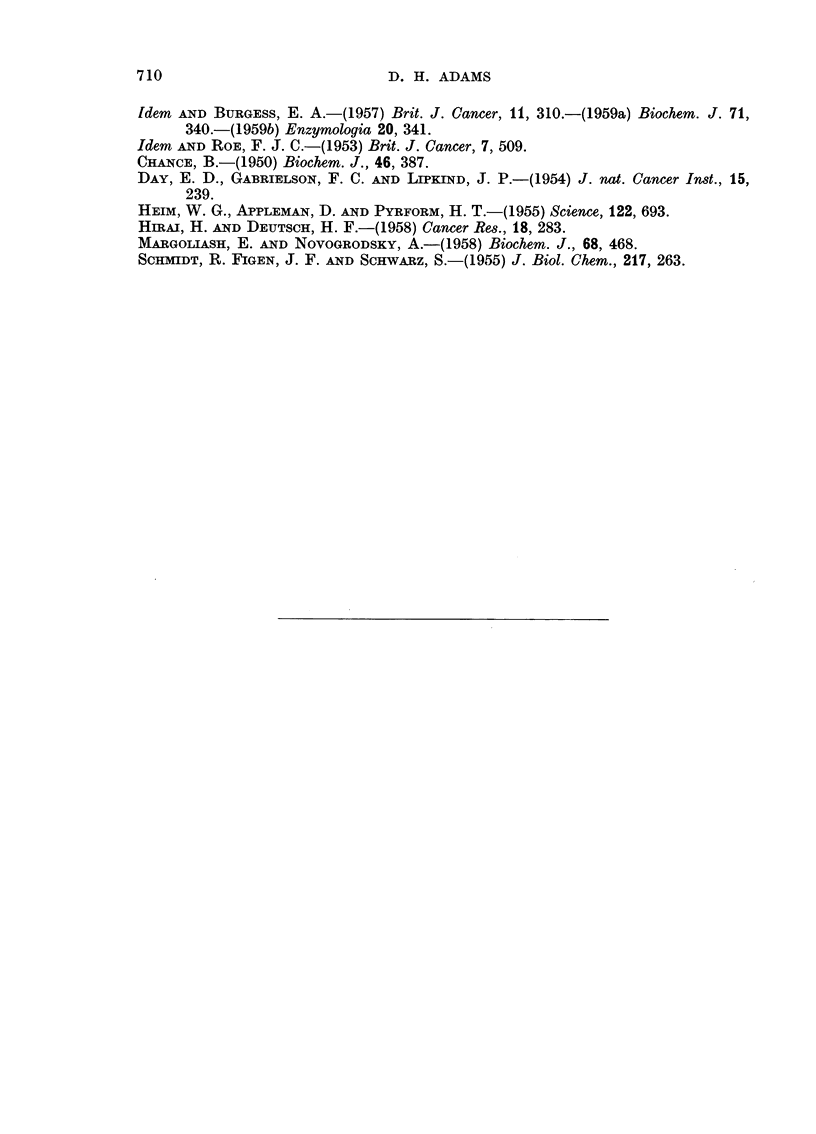# The Effect of Sarcoma 37 on the Intracellular Distribution of Mouse Liver Catalase

**DOI:** 10.1038/bjc.1959.79

**Published:** 1959-12

**Authors:** D. H. Adams


### THE EFFECT OF SARCOMA 37 ON THE INTRACELLULAR DISTRIBUTION OF MOUSE LIVER CATALASE

D. H. ADAMS

*From the Cancer Research Department, London Hospital Medical College, London, E.*1

Received for publication October 23, 1959

MUCH work has been published on the effect on liver catalase of tumour growth, and of the injection of whole or semi-purified tumour homogenates, but in all these studies the sole criterion of activity has been a simple depression in catalase level. An increasingly large number of substances apparently unrelated to tumour tissue, have been shown to depress liver catalase activity on injection, e.g. methyl *bis* (B-chlorethyl) amine, 9 : 10-dimethyl-1 : 2-benzanthracene, butter yellow and its 3'methyl demative, 4'-amino-2 : 3-dimethylaminoazobenzene, B-naphthylamine, benzpyrene (Adams and Roe, 1953), homogenised normal spleen tissue (Day, Gabrielson and Lipkind, 1954), aminotriazole (Heim, Appleman and Pyrform, 1955) allyl isopropyl acetyl carbamide (Schmidt, Figen and Schwarz, 1955), cysteine and a number of its derivatives (Hirai and Deutsch, 1958), thiourea (Margoliash, 1958, personal communication), testosterone and 17-methyl testosterone (Adams, 1960).

There is little direct evidence about the mechanism by which these substances depress catalase activity *in vivo*. Probably none of them directly inhibit the enzyme. Amino triazole acts *in vitro* by combining with catalase-hydrogen peroxide Complex I (Margoliash and Norogrodsky, 1958). However this substance is characterised *in vivo* by an extremely rapid action—liver catalase activity being reduced almost to zero in a few hours. It is well known that hydrogen peroxide destroys catalase and therefore one possible explanation is that these substances affect catalase activity through the production of hydrogen peroxide during their metabolism. Certainly if every substance which gives rise to $H_2O_2$ *in vivo* depresses catalase activity, such depressions are completely non-specific. Has then tumour growth or tumour tissue any specific action on liver catalase ?

Adams and Burgess (1959*a*) recently showed that when liver slices were incubated *in vitro* in a phosphate saline medium, catalase migrated from the large granules into the extra particulate cytoplasm (EPC). This observation led to the suggestion (Adams, 1960) that such catalase migration may occur *in vivo* and that catalase is synthesised only in the large granules. On this view the EPC level would be maintained by catalase derived from the granules by migration through the granule membranes. If this is correct, then the permeability of the granule membranes will be one of the factors determining the EPC catalase level. Adams (1960) also suggested that, in the simplest analysis, the granule/EPC catalase distribution ratio would provide an approximate index of granule permeability. He showed that after the injection of cysteine, methyl butter yellow, or thiourea, depressions in catalase activity were observed in both granule and EPC

fractions, but that these changes occurred without alteration in the granule/EPC distribution ratio. Further evidence in the same paper showed that androgenic hormones altered the granule/EPC distribution ratio in a way consistent with the supposition that they act by increasing the permeability of the large-granule membranes to catalase. Adams (1951) found that there appeared to be an antagonism between the action of testicular and adrenal hormones, and that of tumour homogenate or catalase activity.

The purpose of this paper is to make a preliminary study of the action of tumour growth and tumour homogenate on intracellular catalase distribution.

### MATERIALS AND METHODS

*Animals.*—Young adult mice of the 101 and CBA strains and of an albino strain derived from AKR were used. These animals were all bred in this laboratory by brother-sister mating, but some AKR-substrain mice were obtained direct from the Laboratory Animal Bureau.

*Preparation of liver fractions and estimation of liver catalase activity.*—Catalase estimations were made on whole liver homogenates, and on granule and EPC fractions. The method of estimation of catalase activity has been fully described in previous publications (Adams, 1950, 1952) and the preparation of the homogenates and fractions by Adams and Burgess (1957, 1959*a*). Ethanol (final concentration 0·01 M.) was added to all catalase containing solutions to prevent loss of catalase due to " Complex II " formation (Chance, 1950 ; Adams and Burgess, 1959*b*).

*Tumour.*—Sarcoma 37 was obtained originally from the Imperial Cancer Research Fund Laboratories and maintained by serial passage in this laboratory.

*Triton* × 100.—This non-ionic detergent (kindly given to us by Charles Lennig & Co.) was used at a final concentration of 0·25 per cent v/v to disrupt large granules and liberate their catalase activity into solution.

### RESULTS

### *The injection of normal tissue*

Adams (1950), working with homogenates containing principally EPC fraction, found that the injection of 50 mg. doses of normal tissue did not depress catalase activity significantly. In view of the finding by Day *et al.* (1954) that injections of homogenised spleen did depress liver catalase activity, the effect of larger doses of normal tissue on total, granule, and EPC catalase was investigated. The normal tissue used was a mixture of the liver, spleen, and kidneys taken from sufficient CBA mice to provide the total required. The mixed tissues were homogenised (50 strokes with a Ten Broeck grinder) and injected subcutaneously in 100 and 200 mg. doses into AKR-substrain female mice. As shown in Fig. 1 the lower dose produced only a slight depression in catalase activity, but the higher dose resulted in considerable depression in catalase activity in both granule and EPC fractions. However, the granule/EPC catalase distribution ratio remained almost unaltered through the course of the experiment. Fig. 1 also shows that the effect of a single injection of homogenised rat liver (100 mg.) was similar to that produced by the same dose of mouse tissue. Closely similar results, i.e. depression in catalase activity with little or no alteration in the distribution ratio, were obtained after the injection of cysteine, thiourea, and butter yellow (Adams, 1960).

*The injection of tumour tissue*

Fig. 2 shows the result of the injection of 100 mg. and 200 mg. of homogenate prepared in a similar way from Sarcoma 37 into AKR-substrain females. As with normal tissue, depressions in catalase activity were observed, but with the

FIG. 1.—The effect of the injection of homogenised normal tissue on the catalase activity and granule/EPC catalase distribution ratio of AKR-substrain ♀ mice. (*a*) and (*b*) 100 mg. and 200 mg. of mixed liver kidney and spleen from CBA mice. (*c*) 100 mg. of similar tissue from albino rats. Injection at 0 days.

△———△ Total catalase. ○———○ Granule catalase.
●———● EPC catalase.

Results are expressed in this and subsequent figures as arithmetic means ± Std. errors of means. Twelve control and eight treated animals/group.
*Ordinate.*—Catalase activity in arbitrary units/mg. N.
*Abscissae.*—Time in days.

difference that the granule/EPC catalase distribution ratio increased considerably. Fig. 3 shows the results of the injection of 100 mg. of normal tissue, and 100 mg. of tumour tissue, into CBA males. The normal tissue resulted in depression in catalase activity without alteration in the granule/EPC distribution ratio (3·3 in normal males). The injection of tumour homogenate resulted in an increase in the ratio to 4·6.

*Tumour growth*

The effect of tumour growth on the catalase activity and granule/EPC distribution ratio in AKR-substrain female and 101 male and female mice is shown

FIG. 2.—The effect of the injection of homogenised S37 tissue on the catalase activity and granule/EPC catalase distribution ratio of AKR-substrain ♀ mice. (a) 100 mg. (b) 200 mg. injected at 0 days.

△———△ Total catalase. ○———○ Granule catalase.
●———● EPC catalase.

*Ordinate.*—Catalase activity in arbitrary units/mg. N.
*Abscissae.*—Time in days.

FIG. 3.—The effect of the injection of homogenised tissue on the catalase activity and granule/EPC catalase distribution ratio of CBA ♂ mice.
(a) 100 mg. liver-kidney-spleen taken from AKR mice.
(b) 100 mg. S37 tissue.

△———△ Total catalase. ○———○ Granule catalase.
●———● EPC catalase.

*Ordinate.*—Catalase activity in arbitrary units/mg. N.
*Abscissae.*—Time in days.

in Fig. 4. Progressive decreases in catalase activity in both granules and EPC were associated with progressive increases in the granule/EPC distribution ratio. In the AKR-substrain mice considerable tumour necrosis was found, but little or none in the 101 mice.

### DISCUSSION

Surprisingly enough perhaps comparatively little work has been done on direct comparison of the effect of the injection of normal and tumour tissue on liver catalase. Adams (1950) (measuring principally EPC catalase) found that

Fig. 4.—Effect of S37 tumour growth on catalase activity and granule/EPC catalase distribution ratio of (a) AKR-substrain ♀ mice. (b) 101 ♀ mice. (c) 101 ♂ mice. The tumour bearing mice were formed into groups of 6–8 animals bearing tumours of similar size and the tumour weights averaged to give the points on the graphs.

△——△ Total catalase. ○——○ Granule catalase.
●——● EPC catalase.

*Ordinate.*—Catalase activity in arbitrary units/mg. N.
Abscissae.—Tumour weight in grams.

the injection of 50 mg. dose of normal tissue did not depress mouse liver catalase significantly, but 50 mg. dose of S37 did. The present work shows clearly that catalase depressions may easily be obtained after the injection of normal tissue, provided that the dose is sufficiently high. This depression of catalase activity by normal tissue injection confirms the result of Day *et al.* (1954). In fact if only *total* catalase activity is considered normal tissue homogenate may depress catalase to the same extent, dose for dose, as tumour homogenate. The effect on the intracellular (granule/EPC) catalase distribution ratio was however quite different. Normal tissue homogenate did not alter this ratio in either sex, and this resembled thiourea, cysteine and methyl butter yellow in this respect (Adams,

1960). On the other hand, tumour homogenate increased the granule/EPC ratio considerably in both sexes, and the same phenomenon was seen during tumour growth. The results suggest that injected tumour tissue has two separate actions on catalase. Firstly, the total enzyme level is decreased, an effect which also occurs with normal tissue, and secondly, superimposed on this, there is an increase in the granule/EPC distribution ratio. This action on the distribution ratio, which is apparently specific, shows itself principally by a decrease in the EPC level. Thus if a comparison is made of the action of tumour homogenate and normal tissue homogenate on the EPC catalase level only, tumour tissue will produce a greater depression compared with normal tissue. This accounts for the results previously reported (Adams, 1950) that 50 mg. tumour homogenate significantly depressed the (EPC) catalase activity whereas no depression was caused by normal tissue homogenate.

The results given here, taken in conjunction with those summarised in the introduction, make it clear that a simple catalase depression is not in the least a specific action of tumour tissue or of tumour growth.

It seems also quite clear that the fractions derived from tumour tissue by various investigators cannot, on the available evidence, be said to contain any specific tumour agent. These fractions have been tested solely on the basis of their ability to depress catalase activity *in vivo* after injection. There are obviously many substances which could be present in varying amounts in tumour tissue and in normal tissue, which will produce a catalase depression when injected in adequate dosage.

Tumour growth resulted in a progressive fall in catalase activity, and a progressive increase in granule/EPC distribution ratio. However the catalase activities in these mice fell to lower levels than those observed after injections of tissue homogenates. This point will be dealt with in a future publication.

Adams (1960) showed that androgenic hormones injected into female mice exerted a specific effect in decreasing the liver catalase granule/EPC distribution ratio from the normal value of about 5·0, to the normal male value of about 3–3. This was interpreted as showing that the hormones increase the permeability of the granule membranes to catalase. On the same view, the increase in distribution ratio caused by tumour growth and tumour tissue would result from a decrease in the permeability of the large-granule membranes to catalase. The antagonistic action of tumour tissue and hormones reported by Adams (1951) appears therefore to result from their opposite effects on granule membrane permeability.

SUMMARY

(1) Depression of liver catalase activity results equally well from injections of S37 homogenate or normal tissue homogenates.

(2) S37 homogenate increases the granule/EPC catalase distribution ratio, but normal tissue does not.

(3) It is concluded that S37 homogenate contains an agent which decreases the permeability of the granule membranes to catalase.

### REFERENCES

ADAMS, D. H.—(1950) *Brit. J. Cancer*, **4**, 183.—(1951) *Ibid.*, **5**, 115.—(1952) *Biochem. J.*, **50**, 486.—(1960) *Ibid.* **74**, 141.

*Idem* AND BURGESS, E. A.—(1957) *Brit. J. Cancer*, **11**, 310.—(1959a) *Biochem. J.* **71**, 340.—(1959*b*) *Enzymologia* **20**, 341.
*Idem* AND ROE, F. J. C.—(1953) *Brit. J. Cancer*, **7**, 509.
CHANCE, B.—(1950) *Biochem. J.*, **46**, 387.
DAY, E. D., GABRIELSON, F. C. AND LIPKIND, J. P.—(1954) *J. nat. Cancer Inst.*, **15**, 239.
HEIM, W. G., APPLEMAN, D. AND PYRFORM, H. T.—(1955) *Science*, **122**, 693.
HIRAI, H. AND DEUTSCH, H. F.—(1958) *Cancer Res.*, **18**, 283.
MARGOLIASH, E. AND NOVOGRODSKY, A.—(1958) *Biochem. J.*, **68**, 468.
SCHMIDT, R. FIGEN, J. F. AND SCHWARZ, S.—(1955) *J. Biol. Chem.*, **217**, 263.